# Quartet-based inference of cell differentiation trees from ChIP-Seq histone modification data

**DOI:** 10.1371/journal.pone.0221270

**Published:** 2019-09-26

**Authors:** Nazifa Ahmed Moumi, Badhan Das, Zarin Tasnim Promi, Nishat Anjum Bristy, Md. Shamsuzzoha Bayzid

**Affiliations:** Department of Computer Science and Engineering, Bangladesh University of Engineering and Technology, Dhaka, Bangladesh; Macau University of Science and Technology, MACAO

## Abstract

Understanding cell differentiation—the process of generation of distinct cell-types—plays a pivotal role in developmental and evolutionary biology. Transcriptomic information and epigenetic marks are useful to elucidate hierarchical developmental relationships among cell-types. Standard phylogenetic approaches such as maximum parsimony, maximum likelihood and neighbor joining have previously been applied to ChIP-Seq histone modification data to infer cell-type trees, showing how diverse types of cells are related. In this study, we demonstrate the applicability and suitability of quartet-based phylogenetic tree estimation techniques for constructing cell-type trees. We propose two quartet-based pipelines for constructing cell phylogeny. Our methods were assessed for their validity in inferring hierarchical differentiation processes of various cell-types in H3K4me3, H3K27me3, H3K36me3, and H3K27ac histone mark data. We also propose a robust metric for evaluating cell-type trees.

## Introduction

Cellular differentiation is one of the key aspects of developmental biology. Cell differentiation is known to be a hierarchical process where totipotent cell-types become more specialized cell-types [1, 2]. Thus, the relationship of cell-types is expected to form a tree-like structure [3, 4]. This hypothetical tree-like relationship of cell-types in ontogeny and phylogeny is called a “cell-type tree” [5, 6].

The changes of gene expression patterns during cellular differentiation are recorded as epigenetic changes in the genome [3, 7]. Epigenetic and transcription factors play a vital role in cell differentiation since all cell-types in an individual organism have the same genome [8–10]. Histone modification creates one important class in epigenetic marks which have been found to vary across different cell-types and play an important role in gene regulation [11]. Histone modifications such as methylation, acetylation, phosphorylation, ubiquitination alter their interactions with the DNA and thereby influence transcription and genomic function [11]. A study of the change in histone marks across various cell-types can help us understand how cell differentiation occurs [12]. Since the development of the sister cell-types is the same up to the last stages of differentiation [1], evolutionary relatedness of cell-types is expected to be congruent with the ontogenetic hierarchy of cellular differentiation [13]. Therefore, constructing and analyzing cell-type trees could play an important role in our understanding of developmental biology and how cell differentiation occurs [6, 14, 15].

The process of elucidating the hierarchical developmental relationships among cell-types depends on extremely laborious experiments involving in vitro differentiation of cell-types from various stem cell-types [16–18]. Fortunately, the propitious advancement in sequencing technologies has enabled us to capture transcriptomic and epigenetic information at various developmental stages [4]. Considering the hypothesis about the tree-like structure of the cell differentiation process, various techniques from phylogenetic tree estimation have been adapted to form cell-type trees by leveraging various sequence data (e.g., ChIP-Seq, RNA-seq).

The application of traditional phylogenetic tree estimation methods (maximum likelihood (ML), maximum-parsimony (MP) and neighbor joining (NJ)) have previously been applied for building cell-type trees [14, 15]. These studies underscore the usefulness of phylogenetic tree estimation techniques for reconstructing the hierarchical process of cell differentiation. ML-based technique was shown to be a better technique than MP and NJ. In this paper, we demonstrate the usefulness of quartet-based tree estimation methods in the context of cell-type trees. Quartet based phylogenetic tree estimation is very popular and highly accurate since quartet is a statistically consistent estimate of the true species phylogeny despite gene tree heterogeneity due to the presence of incomplete lineage sorting [19, 20]. Quartet based methods are robust to the “anomaly zone” [20, 21] (a condition where there could be gene tree topologies that are more likely than the one that has the same topology as the species tree) as there are no anomalous unrooted four-taxon species trees [19, 20]. Thus, various quartet-based techniques have been developed and are being widely used due to their excellent accuracy [22–26].

In this study, we attempt to leverage the theoretical and practical advantages of quartet-based techniques in constructing the cell-type trees. We propose two pipelines for inferring cell-type trees: 1) *Induced Quartet Amalgamation* (IQA), and 2) *Most Likely Quartet Amalgamation* (MLQA). Both these pipelines start with estimating quartets (in two different ways) from ChIP-Seq data and then amalgamate the quartets to construct cell-type trees. We performed an extensive experimental study using H3K4me3, H3K27me3 and H3K36me3 histone modification data, and compared our techniques with ML-based technique. Unlike previous studies, we included both the normal and cancerous cell-types to examine the power and applicability of phylogenetic methods in analyzing both kinds. We also proposed a new evaluation criterion to evaluate the cell-type trees which is more robust than the evaluation metrics used in previous studies [14, 15], especially in the presence of “alien” cell-types within a cluster of a particular cell-type. This can happen either because the data do not have enough phylogenetic information to clearly distinguish the cell-types or due to the presence of “rogue taxa”—some taxa that are relatively unstable in phylogenetic analyses [27–29]. Rogue taxa assume varying phylogenetic positions in a collection of trees and thus have negative impact in phylogenetic analyses, especially in estimating consensus history [28, 29]. Finally, we conclude that quartet-based phylogenetic tree estimation can be considered as a useful and robust technique for inferring cell-type trees.

## Materials and methods

### Data preprocessing

Histone marks are found in every 200 base pair length of DNA [15]. ChIP-Seq is a technology which records histone modification throughout the whole genome. It is assumed that histone marks can be independently gained or lost in regions of the genome during cell differentiation [6].

ChIP-Seq data are converted into peak data where the peak signifies presence of histone marks in the genome. Similar to previous studies [6], we used peak lists as the raw data for our study. We represent the data based on the presence or absence of peaks at any given position and treat this as a binary character. One can use any peak finder, such as MACS (Model-based analysis of ChIP-Seq [30]), PeakSeq [31], Hotspot [32], to convert the ChIP-Seq histone modification libraries into peaks. We used the publicly available peaks given by the ENCODE project (for H3K4me3, H3K27me3, H3k36me3) and CISTROME DB [33] (for H3K27ac) in our analysis. Previous studies [6] introduced two different data representations: 1) *Windowing* representation and 2) *Overlap* representation. In windowing representation, a ChIP-Seq library (a cell-type) is divided into bins of certain sizes, and each of the bins are coded as either 1 or 0 depending on whether there exist at least one peak in a bin. In overlap data representation, all ChIP-Seq libraries are taken into account at once and “interesting regions” based on genome peaks are identified (see [6] for details). Considering each peak as an interval on the genome, the *interval graph* is defined by all peaks in all libraries. An interval graph has one vertex for each interval and an edge between two vertices when the corresponding intervals overlap [34]. With these representation techniques, ChIP-Seq libraries are represented as strings of 0s and 1s. In this study, we used the overlap representation since no notable difference was found between these two techniques in terms of the reliability of the cell-type trees, and overlap representation was preferred in previous studies for its compactness [6, 15].

We wrote necessary scripts in C++, Perl and Python to implement our proposed methods (available at https://github.com/Moumiiiiii/cell-differentiation-trees). The scripts for overlap representation was obtained from the authors (Nair *et al*. [6]). We used QFM [23] to amalgamate quartets (as described in the following section). The code for QFM was obtained from the authors (Reaz *et al*. [23]).

### Overview of the quartet-based pipelines

**Fig 1:**
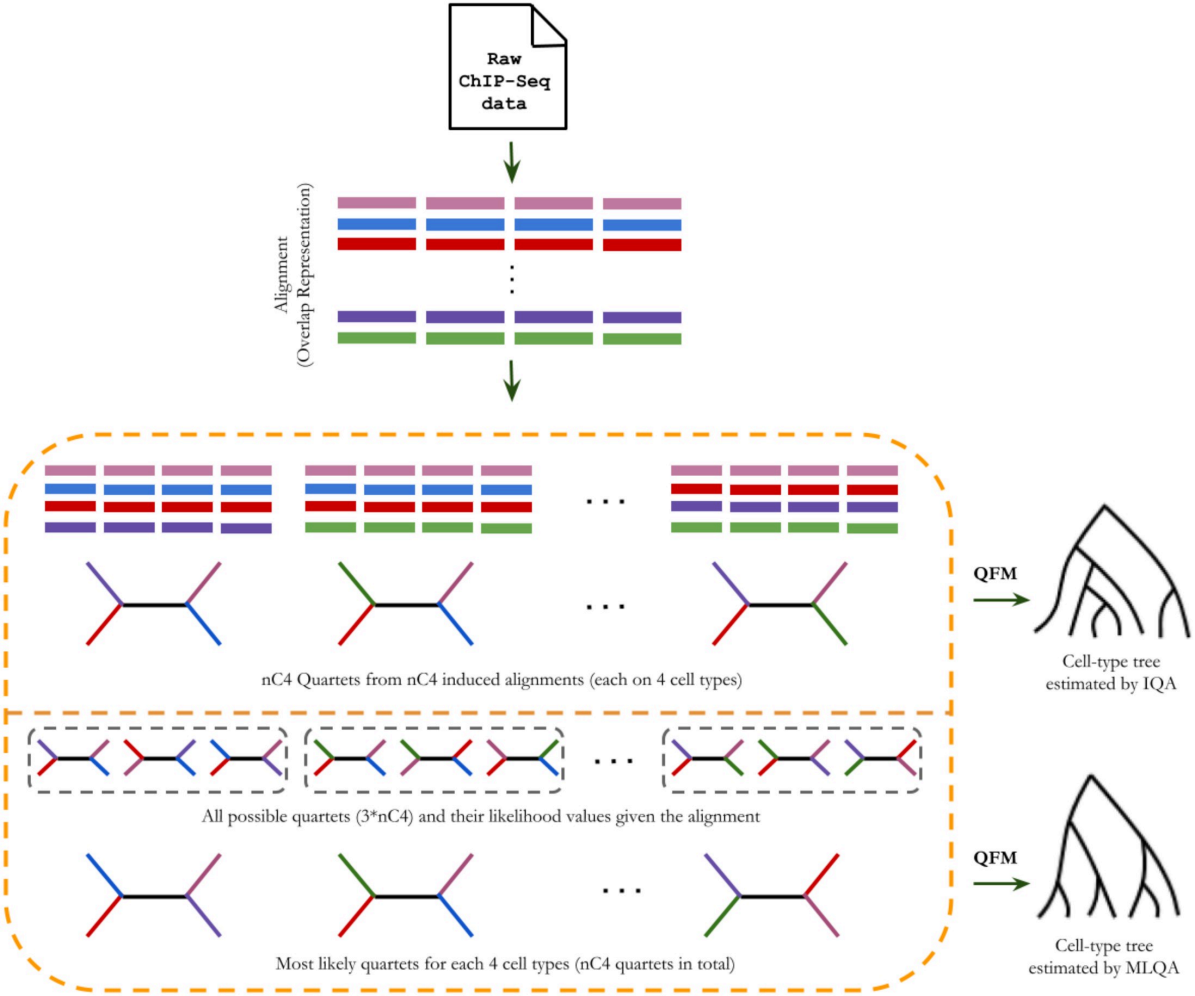
Illustration of the methodology for quartet-based cell-type tree construction. We begin with the binary data matrix resulted from the ChIP-Seq data libraries using the overlap representation. Next, we generate a set of $\left(\begin{array}{c} n\\ 4 \end{array}\right)$ quartets in two different ways. Finally, we amalgamate these quartets using QFM [23].

Quartet is an unrooted tree with four taxa. We denote a quartet by *q* = *ab*|*cd*, where the internal edge in *q* separates *a* and *b* from *c* and *d* (meaning that *ab*|*cd* is the bipartition defined by the internal edge in *q*). Unlike previous studies [6, 15] that used maximum-parsimony, maximum-likelihood or neighbor joining method on the binary data matrix obtained from the ChIP-Seq libraries, we estimate quartets—representing the evolutionary history of four cell-types. For a collection of *n* cell-types, we estimate a set of $\left(\begin{array}{c} n\\ 4 \end{array}\right)$ quartets (one for each group of 4 cell-types). We generate this set of $\left(\begin{array}{c} n\\ 4 \end{array}\right)$ quartets in two different ways (referred to as IQA and MLQA, and are described in subsequent sections). Finally, we amalgamate these quartets to get a single coherent tree on *n* cell-types. A quartet *q* is consistent with a tree when the tree has an internal edge that separates the same pairs of taxa as in *q*. Note that it may not always be possible to find a tree which is consistent with all the $\left(\begin{array}{c} n\\ 4 \end{array}\right)$ quartets. In that case, we try to find a tree such that maximum number of quartets are consistent with it. This is an NP-hard problem [35], but efficient methods such as QFM [23] and QMC [24] are available for quartet amalgamation. We used QFM since it was shown to have better accuracy compared to QMC [23]. However, QMC is faster than QFM and we recommend QMC in case the dataset is too large for QFM to analyze. Fig 1 illustrates our proposed methodologies for quartet-based cell-type tree construction.

#### Induced Quartet Amalgamation (IQA)

Given a binary data matrix *M* on *n* cell-types obtained from the overlap representation, we consider all possible combinations of 4 cell-types and thus have a collection of $\left(\begin{array}{c} n\\ 4 \end{array}\right)$ groups. For each group of 4 cell-types, we generate a data matrix *M*_*i*_ (1 ≤ *i* ≤ $\left(\begin{array}{c} n\\ 4 \end{array}\right)$) which contains four rows corresponding to the four cell-types. Next, for each *M*_*i*_, we estimate a quartet using maximum-likelihood approach. For this purpose, we used RAxML [36]. Thus we have a set of $\left(\begin{array}{c} n\\ 4 \end{array}\right)$ induced quartets from *M*. Finally, we combine these quartets using QFM to estimate a tree on *n* cell-types.

#### Most Likely Quartet Amalgamation (MLQA)

For each group of 4 taxa (*a*, *b*, *c*, *d*), there are three different quartet topologies: ((*a*, *b*), (*c*, *d*)), ((*a*, *c*), (*b*, *d*)) and ((*a*, *d*), (*b*, *c*)). Thus, there are $3*(\begin{array}{c} n\\ 4 \end{array})$ possible quartets on *n* cell-types. In MLQA, we generate all these quartets and their associated likelihood values (with respect to *M*) using RAxML. Next, from the three different quartet topologies on four cell-types, we retain the one which has the highest likelihood value. In this way we have a collection of $\left(\begin{array}{c} n\\ 4 \end{array}\right)$ quartets. Finally, we combine these most likely quartets using QFM to get a single cell-type tree.

### Experimental studies

#### Dataset

Histone modification ChIP-Seq data was collected from the ENCODE database [37]. We used H3K4me3, H3k27me3, H3k36me3 and H3K27ac dataset. The histone modification peak data was obtained from the ENCODE database and CISTROME DB [33]. Although the differentiation process of cancerous cell-types differ from normal cell-types, we considered both normal and cancerous cell-types to determine how phylogenetic approaches perform under various cell differentiation processes. All the cell-types used for the experimentation process are listed in Table 1. We used two replicates for most of the cell-types except for HCFaa, HFF, and CD14 since the ENCODE database contains only one replicate for these cell-types. We have data from different timestamps (day 0, 2, 5, 9, 14) of differentiation process for human Embryonic Stem cell-types (hESC). So we denote by H7_hESC_T5 the data from day 5.

**Table 1:** Cell-types, short description, and general group for H3K4me3, H3K27me3, H3K36me3, and H3K27ac data.

Cell name	Short description	Group	Tissue type	H3K4me3	H3K27me3	H3K36me3	H3K27ac
7250(Hs352.Sk)	Unavailable	Fibroblast	Skin, muscle	-	-	-	✓
AG04449	fetal buttock/thigh fibroblast	Fibroblast	Skin	✓	-	-	-
AG04450	fetal lung fibroblast	Fibroblast	Lung	✓	✓	-	✓
AG09319	gum tissue fibroblasts	Fibroblast	Gingival	✓	-	-	-
AoAF	aortic adventitial fibroblast cell-types	Fibroblast	Blood vessel	✓	-	-	-
BJ	skin fibroblast	Fibroblast	Skin	✓	✓	✓	✓
CACO2	human colorectal adenocarcinoma cell-types	Epithelial	Colon	-	✓	✓	-
CD14	Monocytes-CD14+ from human leukapheresis production	Blood	Blood	✓	✓	-	-
CD20(1)	B cell-types replicate, African American	Blood	Blood	✓	-	-	-
CD20(2)	B cell-types replicate, Caucasian	Blood	Blood	✓	-	-	-
GM06990	B-lymphocyte	Blood	Blood	-	✓	✓	-
GM12873	B-lymphocyte, lymphoblastoid	Blood	Blood	-	-	-	✓
GM12878	B-Lymphocyte	Blood	Blood	-	✓	✓	✓
GM18526	lymphoblastoid	Blood	Blood	-	-	-	✓
GM19240	B-lymphocyte, lymphoblastoid	Blood	Blood	-	-	-	✓
HAc	astrocytes-cerebellart	Astrocytes	Cerebellar	✓	-	-	-
HAsp	astrocytes spinal cord	Astrocytes	Spinal cord	✓	-	-	-
HBMEC	brain microvascular endothelial cell-types	Endothelial	Blood vessel	✓	-	-	-
HCC827	lung cancer cell	Epithelial	Lung	-	-	-	✓
HCF	cardiac fibroblast	Fibroblast	Heart	✓	-	-	-
HCFaa	cardiac fibroblasts- adult atrial	Fibroblast	Heart	✓	-	-	-
HCM	cardiac myocytes cell	Myocytes	Heart	✓	-	-	-
HCPEpiC	choroid plexus epithelial cell-types	Epithelial	Epithelium	✓	-	-	-
HCT-15	quasidiploid human cell line	Epithelial	Colon	-	-	-	✓
HEEpiC	esophageal epithelial cell-types	Epithelial	Epithelium	✓	-	-	-
HEK293T	highly transfectable derivative of human embryonic kidney 293 cells	Epithelial	Kidney	✓	-	-	-
Hela-S3	cervical adenocarcinoma	Epithelial	Cervix	-	✓	✓	✓
HepG2	human liver cancer cell line	Epithelial	Liver	-	✓	✓	✓
hESC	undifferentiated embryonic stem cell-types	hESC	Embryonic stem cell	✓	✓	✓	-
HFF	foreskin fibroblast	Fibroblast	Foreskin	✓	-	-	-
HFF MyC	foreskin fibroblast cell-types expressing canine cMyc	Fibroblast	Foreskin	✓	-	-	-
HMEC	mammary epithelial cell-types	Epithelial	Breast	✓	✓	-	-
HPAF	pulmonary artery fibroblasts	Fibroblast	Blood vessel	✓	-	-	-
HPF	pulmonary fibroblasts isolated from lung tissue	Fibroblast	Lung	✓	-	-	-
HRE	renal epithelial cell-types	Epithelial	Epithelium	✓	✓	✓	-
HRPEpiC	retinal pigment epithelial cell-types	Epithelial	Epithelium	✓	-	-	-
Huh7	well differentiated hepatocyte-derived carcinoma cell line	Epithelial	Liver	-	-	-	✓
HUVEC	umbilical vein endothelial cell-types	Endothelial	Blood vessel	✓	✓	✓	-
HVMF	villous mesenchymal fibroblast cell-types	Fibroblast	Connective	✓	-	-	-
IMR90	fetal lung fibroblasts	Fibroblast	Lung	-	-	-	✓
JHU-06	cancer cell line	Endothelial	Blood	-	-	-	✓
JHU-11	cancer cell line	Blood	Blood	-	-	-	✓
K562	human myelogenous leukemia cell	Blood	Blood	-	✓	✓	-
KOPT_K1	lymphoma or leukaemia cancer cell line	Blood	Blood	-	-	-	✓
LCL	lymphoblastoid	Blood	Blood	-	-	-	✓
MCF-10A	mammary gland, non-tumorigenic epithelial, inducible cell line	Epithelial	Breast	-	-	-	✓
NHDF Neo	neonatal dermal fibroblasts	Fibroblast	Skin	✓	-	-	-
NHEK	epidermal keratinocytes	Epithelial	Skin	✓	✓	✓	-
NHLF	lung fibroblasts	Fibroblast	Lung	✓	-	-	✓
RPTEC	renal proximal tubule epithelial cell-types	Epithelial	Epithelium	✓	-	-	-
SAEC	small airway epithelial cell-types	Epithelial	Epithelium	✓	✓	✓	-
SKMC	skeletal muscle cell-types	Skeletal Muscle	Brain	✓	-	-	-
SKNMC	human neuroblastoma cell	Epithelial	Brain	✓	-	-	-
SKNSH	human Neuroblastoma Cell	Epithelial	Brain	-	✓	✓	-
TAM_R	human breast cancer cell line	Epithelial	Breast	-	-	-	✓
Toledo	lymphoblastoid	Blood	Blood	-	-	-	✓
WI_38	embryonic lung fibroblast cells	Fibroblast	Embryonic lung	✓	-	-	-
WI_38_TAM	embryonic lung fibroblast	Fibroblast	Embryonic lung	✓	-	-	-

#### Evaluation criteria

To evaluate the estimated cell-type trees, total number of cell-types in a subtree that belong to a particular group was considered by Nair *et al*. [6, 15]. Since the cell-types within a particular group (e.g., Fibroblast, Epithelial, etc.) can be scattered across multiple subtrees, the two largest subtrees were considered for each cell-type. The larger this quantity is for a certain approach, the better its performance is for that particular cell-type. We note that this metric tends to be very sensitive towards a single intrusion of an alien cell-type of a different group within a subtree since it considers the clades containing only a particular group of cell-types. Therefore, we have introduced a new metric (*α*), which takes the relative abundance of a particular group of cell-type in a clade compared to the other groups of cell-types. A formal definition of the *α* ratio is as follows.

$$\begin{array}{c} \alpha =\frac{\text{number}\;\text{of}\;\text{cell-types}\;\text{that}\;\text{belong}\;\text{to}\;\text{the}\;\text{same}\;\text{group}\;\text{in}\;\text{a}\;\text{subtree}}{\text{size}\;\text{of}\;\text{that}\;\text{subtree}} \end{array}$$

**Fig 2:**
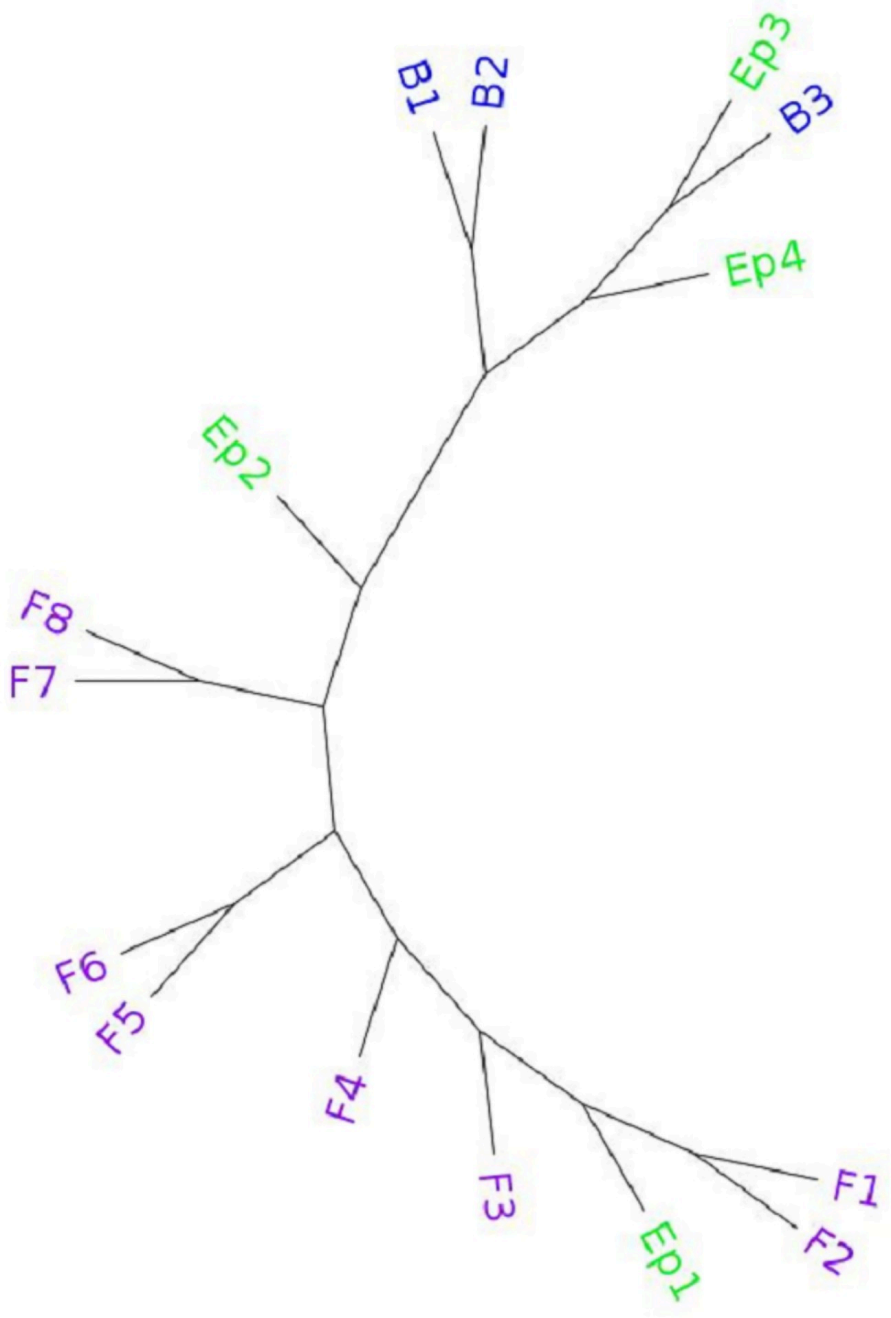
Example for *α* ratio. One Eplithelial (EP1) cell-type has been placed within a subtree containing all the Fibroblast cell-types (*F*_1_ ∼ *F*_8_). Thus the largest subtree containing only Fibroblast cell-types is 2.

Higher values of *α* ratio indicate better clustering of the same cell-types (*α* = 1 indicates that there is a clade that contains *only* the cell-types within a particular group). This evaluation metric is comparatively more tolerant towards an intrusion of alien cell-types within a cluster of a particular cell-type. For better understanding of our proposed *α* ratio, we have shown an example in Fig 2. The cell-type tree in this figure contains three different groups of cell-types (*F*_1_ ∼ *F*_8_, *Ep*_1_ ∼ *Ep*_4_, and *B*_1_ ∼ *B*_3_). For the eight Fibroblast cell-types, the two largest clades containing only Fibroblast cell-types are of size 2 ((*F*_1_, *F*_2_) and (*F*_7_, *F*_8_)). Thus, according to the metric used in previous studies, the evaluation measure for the Fibroblast cell is (2,2). This result gives a misleading impression of the Fibroblast cell-types being scattered sparsely. This happened because of the intrusion of a single Epithelial cell within a clade that contains all the eight Fibroblast types. On the other hand, the *α* ratio for this cell-type is $\frac{8}{9}$, implying that all the eight Fibroblast cell-types were contained in a subtree with nine cell-types. So the *α* ratio rightly shows that the result is not as bad as the first metric indicated.

Thus, the *α* ratio along with the previous metric used in [6] can better elucidate the relative accuracy of various estimated cell-type trees. We have considered this ratio for groups with substantial numbers of cell-types (mostly Fibroblast and Epithelial), since for the other groups with small numbers of cell-types, *α* ratio may not be required to get a better understanding of the relative performance.

## Results and discussion

### Results on H3K4me3 dataset

Trimethylation of Histone H3 at Lysine 4 (H3K4me3) is a well studied histone mark which is associated with transcription start sites of active genes [38]. We analyzed both replicate 1 and 2 for H3k4me3 dataset. Replicate 1 includes 37 cell-types and replicate 2 includes 34 cell-types of 8 different groups. We did not consider both the replicates together as the combined dataset becomes prohibitively large (in terms of computational time and space complexity) to analyze as we have to consider $3*(\begin{array}{c} 71\\ 3 \end{array})$ quartets. This dataset does not contain any cancerous cell-type.

#### Replicate 1

**Fig 3:**
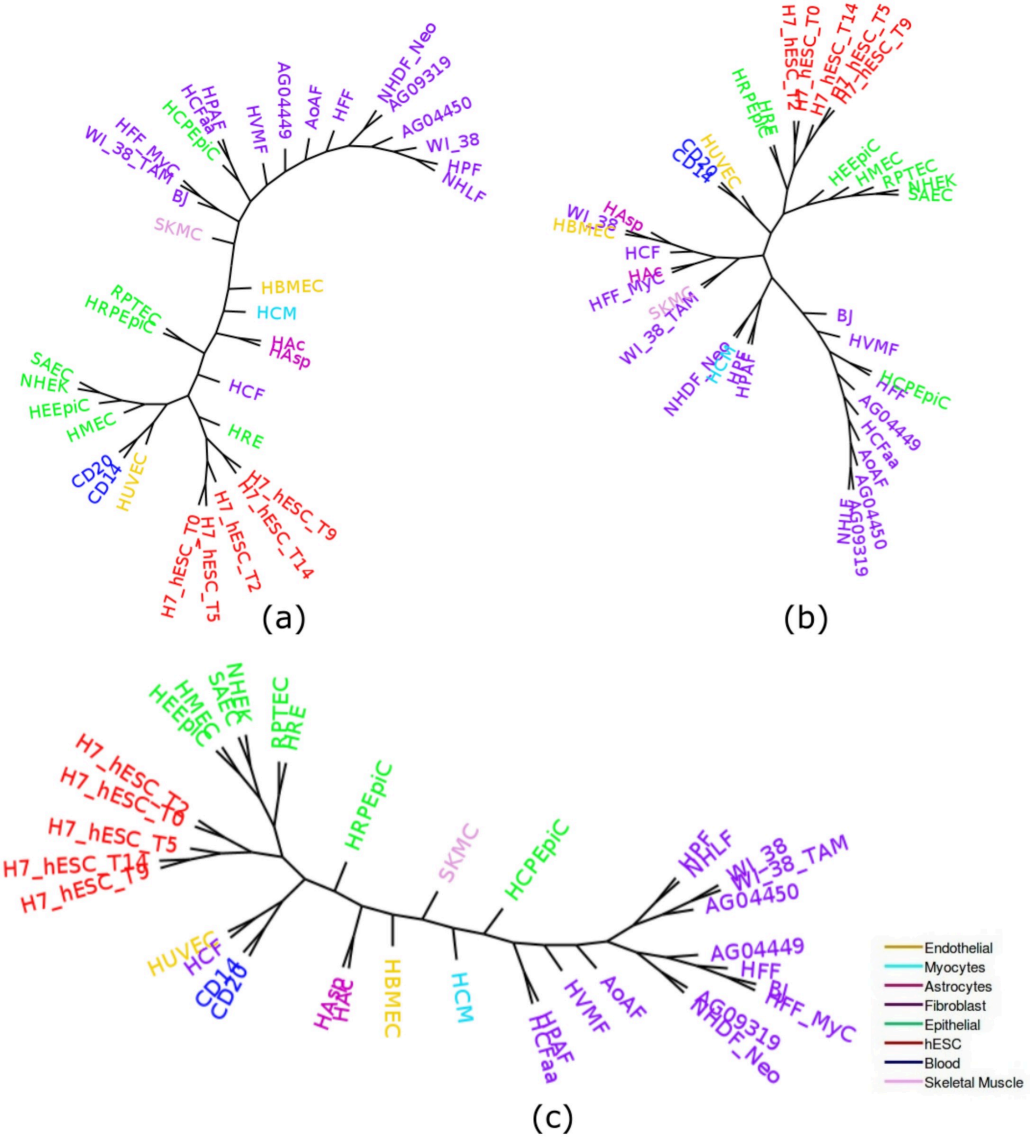
Cell-type trees on H3K4me3 (replicate 1). (a) IQA approach, (b) MLQA approach, and (c) ML approach.

Cell-type tree was constructed using overlap representation from the histone mark of the 37 cell-types. Fig 3 shows the trees constructed by three approaches (ML, IQA and MLQA) with color coding to clearly differentiate various groups of cell-types. In general, we can observe that similar types of cell-types tend to form a clade. For example, all the hESC cell-types are placed inside a single subtree and are clustered together. Moreover, even within this subtree in IQA tree, cell-types from day 9 and day 14 are clustered together and are separated from the subtree that includes day 0, 2 and 5; whereas cell-types from day 5, 9, 14 and cell-types from day 0, 2 are clustered separately in two adjacent subtrees in MLQA approach and ML based approach.

Table 2 shows the number of cell-types belonging to the largest and the second-largest clusters for a particular cell-type. Ideally one group should include all of the cell-types and the other should have zero member (just like hESC group) to exhibit their tendency in clustering together. However, it is not necessarily the case for some of the cell-types. While hESC, Skeletal muscle, Blood, Myocytes, and Astrocytes were clustered ideally for IQA approach, all of the 8, 2 and 16 Epithelial, Endothelial and Fibroblast cell-types were not clustered together. Likewise, in MLQA approach, we can observe ideal clustering for hESC, Skeletal muscle, Blood and Myocytes. But it failed to cluster 2 cell-types from Astrocytes. But it is noteworthy from Fig 3 that, a single Epithelial cell (HCPEpiC) has entered within the Fibroblast cluster in IQA approach, in absence of which, IQA tree would have a (15,1) pair for this dataset. Similar trends hold for MLQA and ML trees. In MLQA tree, HCPEpiC has been placed within a cluster of Fibroblast cell-types, and in ML tree HCPEpiC is not clustered with other Epithelial cell-types.

**Table 2:** Groupings for cell-type trees on H3K4me3 (replicate 1) data using various phylogenetic approaches.

	hESC (5)	Skeletal Muscles (1)	Blood (2)	Myocytes (1)	Astrocytes (2)	Epithelial (8)	Endothelial (2)	Fibroblast (16)
IQA	(5,0)	(1,0)	(2,0)	(1,0)	(2,0)	(4,2)	(1,1)	(10,3)
ML	(5,0)	(1,0)	(2,0)	(1,0)	(2,0)	(6,1)	(1,1)	(15,1)
MLQA	(5,0)	(1,0)	(2,0)	(1,0)	(1,1)	(5,2)	(1,1)	(6,2)

**Fig 4:**
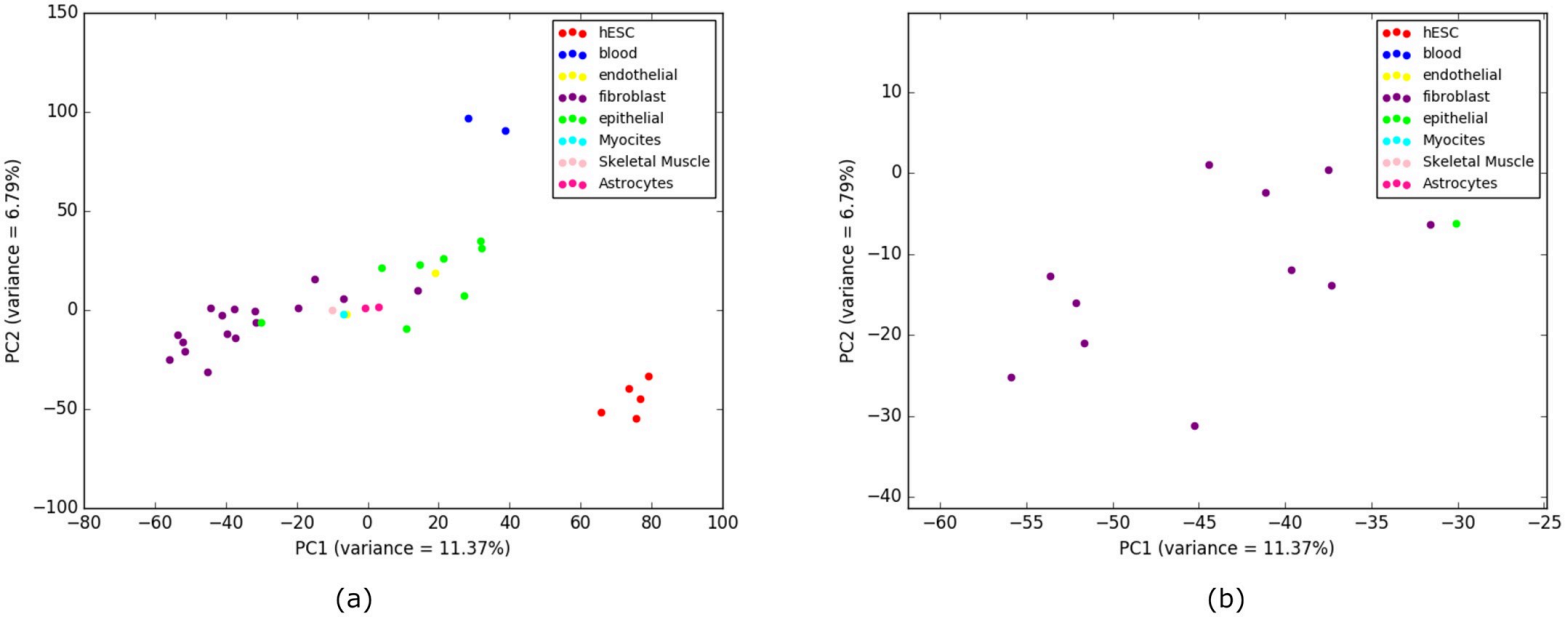
PCA performed on H3K4me3 (replicate 1) and corresponding scores are plotted on PC1 and PC2. (a) 9 closely clustered cell-types that include 8 Fibroblast cell-types and one alien Epithelial cell-type are selected from the 37 cell-types in a rectangular box. It is notable that this intruder Epithelial cell-type (HCPEpiC) is the same one from the cluster of 15 Fibroblast cell-types in the tree (Fig 3) generated using IQA approach. This observation reflects a deeper similitude between the cell-type tree using IQA based approach and the PCA for this dataset. (b) 9 cell-types from (a) are zoomed in for a comprehensible view.

In order to investigate this unstable placement of some cell-types, we performed Principal Component Analysis (PCA) [39] on the overlap representation of the histone modification data. PCA reduces the dimensionality of the data while retaining most of the variation in the dataset by identifying directions, called principal components, along which the variation in the data is maximal [39]. In the PCA plot in Fig 4, we plotted the cell-types along PC1 and PC2 (two principal components that captures the largest and the second largest amounts of variance). PCA analysis reflects that the histone mark data of HCPEpiC is indeed more closely related to the Fibroblast cell-types than it is to the other Epithelial cell-types. PCA analysis also explains the placement of two Endothelial cell-types in two separate clusters as the PCA plot clearly shows that these two cell-types are not closely related according to the data from histone modification. Moreover, PCA analyses support the groupings of the hESC and Blood cell-types (as recovered by most of the the phylogenetic approaches) without the intrusion of any alien entity. Therefore, phylogenetic methods are able to capture the variability in the histone modification data.

We now compare the trees in terms of the *α* ratio (see Table 3). We considered *α* ratio for two cell groups: Epithelial and Fibroblast. Since the other cell-types in different groups are clustered together or lacks substantial numbers of cell-types in them, they are not considered for being analyzed with the *α* ratio. For each cell group, we first show the *α* ratio for the largest subtrees that contain only the cell-types of a particular group. Then we gradually increase the number of cell-types from that group and consider the smallest clade required to house those numbers of cell-types. The largest clades in the ML, MLQA and IQA trees that contain only cell-types from Epithelial group have 6, 5, and 4 cell-types, respectively. Next, as we increase the number of cell-types, it takes a subtree of 13 cell-types for IQA to accommodate 5 Epithelial cell-types. Next, to house 7 cell-types, IQA needs a subtree of 16 entities. Finally, to include all the 8 Epithelial cell-types, it takes a subtree of 27 cell-types. Similarly, we show the *α* ratio for the three approaches for both Epithelial and Fibroblast groups. MLQA approach takes a subtree with 29 cell-types to accommodate 8 Epithelial cell-types whereas ML based method takes a subtree of 22 cell-types and IQA approach takes a subtree with 27 cell-types. Therefore, the Epithelial cell-types are more closely related in IQA approach and ML based approach than they are in the MLQA based approach. The relevance and strength of the *α* ratio in assessing the quality of cell-type trees is more prominent when we looked at the Fibroblast cell-types. Although MLQA seemed to be performing very poorly on Fibroblast group (see Table 2) with (6,2) groupings compared to the ML-based technique with (15,1) groupings, we can see that MLQA takes a subtree of 22 cell-types to cluster all the 16 Fibroblast cell-types whereas ML tree takes 26 cell-types. Similar trend holds for IQA which takes 16 cell-types to house 15 Fibroblast cell-types (just one more than the ML tree), but reconstructs a clade with 24 cell-types (which is 2 less than the ML tree) containing all the 16 Fibroblast cell-types. Thus *α* ratio enables us to evaluate cell-type trees by looking at various numbers of cell-types within a particular cell-type group and gives a better understanding about the relative performance.

**Table 3:** *α* ratio for various cell-type trees on H3K4me3 (replicate 1) data.

	Epithelial (8)	Fibroblast (16)
ML tree	$\frac{6}{6}$, $\frac{7}{16}$, $\frac{8}{22}$	$\frac{15}{15}$, $\frac{16}{26}$
MLQA tree	$\frac{5}{5}$, $\frac{7}{12}$, $\frac{8}{29}$	$\frac{6}{6}$, $\frac{9}{10}$, $\frac{12}{14}$, $\frac{16}{22}$
IQA tree	$\frac{4}{4}$, $\frac{5}{13}$, $\frac{7}{16}$, $\frac{8}{27}$	$\frac{10}{10}$, $\frac{15}{16}$, $\frac{16}{24}$

We also looked at how similar cell-types from different tissue types are related to each other and observed interesting and biologically meaningful relationships. HUVEC (which is an endothelial cell) was placed as a sister to the clade containing CD14 and CD20 (blood type). This placement seems to be biologically meaningful as HUVEC is from blood vessel and CD14 and CD20 are from blood tissue. Another important observation is that, among the fibroblast cell-types, those that are from lung tissue (AG04450, NHLF and HPF) are clustered together. We note that WI_38 and WI_38_TAM—two cell-types from embryonic lung tissue—are sister in the ML tree, but they are not sister in the IQA tree. Interestingly, in the IQA tree, WI_38 was placed as a sister to the clade containing NHLF and HPF that are also from lung tissue. Similarly, the fibroblast cell-types from the skin and foreskin tissues tend to be grouped together. Moreover, cell-types from heart (HCFaa, HCF, HCM) and blood vessel (HPAF, HBMEC) appear to be closely related. Among the epithelial cell-types, those that are from epithelium tissue tend to form a cluster. Moreover, all the cells from embryonic stem cell tissue type are placed within a single clade. Similar trends are observed for other dataset as well, and thus are not detailed in the subsequent sections.

**Fig 5:**
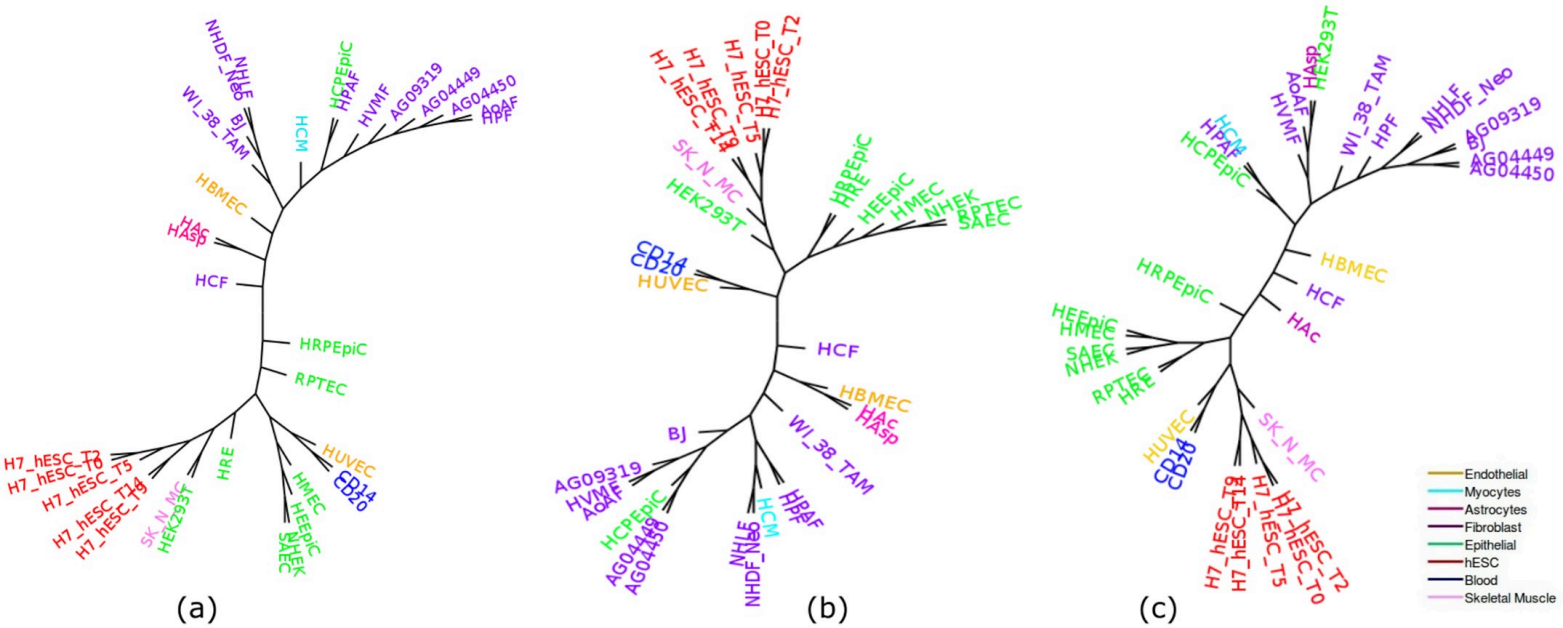
Cell-type trees for H3K4me3 (replicate 2). (a) IQA approach, (b) MLQA approach, and (c) ML approach.

**Fig 6:**
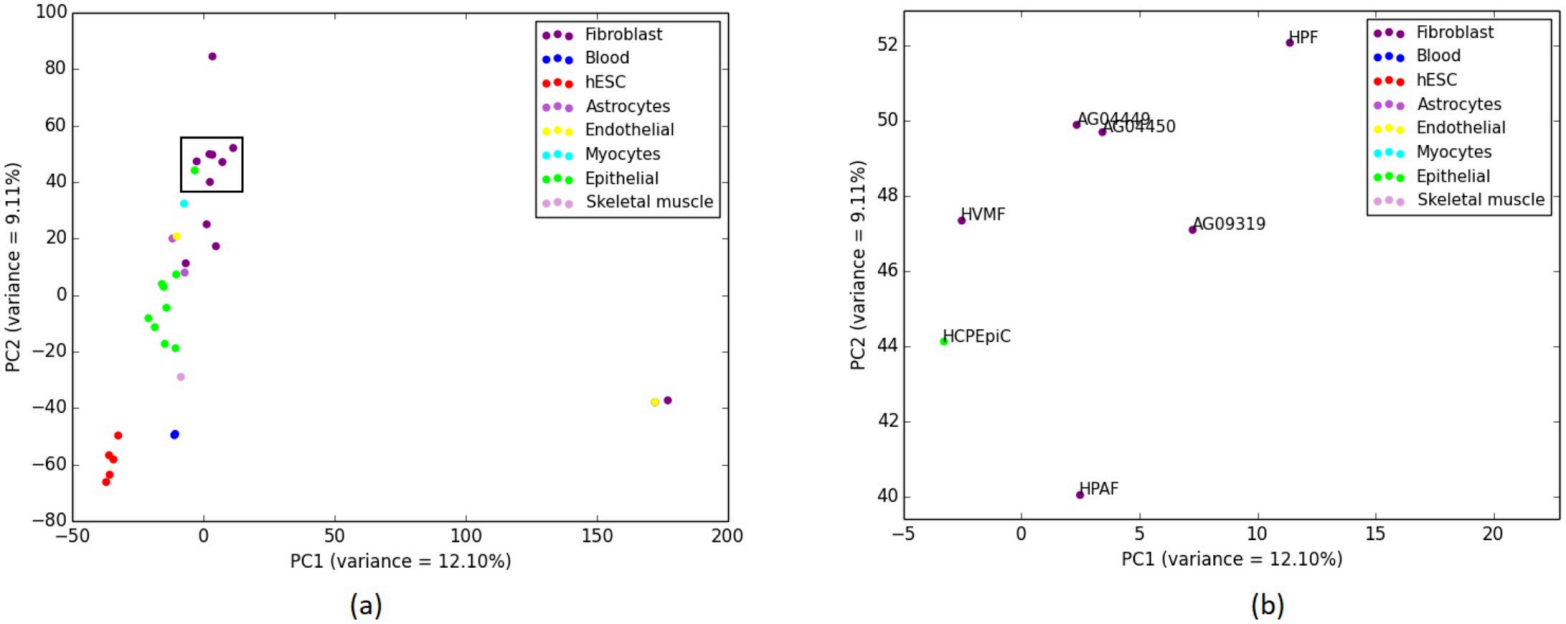
PCA on H3K4me3 (replicate 2) and corresponding scores are plotted on PC1 and PC2. (a) 7 closely clustered cell-types including Fibroblast cell-types and one alien Epithelial cell (HCPEpiC) are selected from the 34 cell-types in a rectangular box. This particular Epithelial cell-type was placed within a subtree containing the Fibroblast cell-types in the cell-type trees estimated by all three methods (see Fig 5). (b) 7 cell-types from (a) are zoomed in for a comprehensible view.

Fig 5 shows the trees constructed by IQA, MLQA and ML on replicate 2. All these trees ideally placed the hESC, Skeletal Muscles, Blood and Myocytes cell-types in separate clusters. In addition to these, IQA and MLQA clustered the Astrocytes cell-types together, where ML tree failed to put them together in a single cluster. ML tree produced better groupings for the 12 cell-types in the Fibroblast group by placing 8 of them in a single subtree (see Table 4). However, if we look at the intermediate *α* values in Table 5, IQA and MLQA are in fact better than ML as they take smaller subtrees to group various numbers of cell-types than ML tree. When we consider all the 12 Fibroblast cell-types, the *α* ratio is same for all these three methods. Similar to replicate 1, we performed PCA analysis which is consistent with the placement of the cell-types in the trees (see Fig 6).

**Table 4:** Groupings for cell-type trees on H3K4me3 (replicate 2) data.

	hESC (5)	Skeletal Muscles (1)	Blood (2)	Myocytes (1)	Astrocytes (2)	Epithelial (9)	Endothelial (2)	Fibroblast (12)
IQA	(5,0)	(1,0)	(2,0)	(1,0)	(2,0)	(4,1)	(1,1)	(6,4)
MLQA	(5,0)	(1,0)	(2,0)	(1,0)	(2,0)	(7,1)	(1,1)	(3,2)
ML	(5,0)	(1,0)	(2,0)	(1,0)	(1,1)	(6,1)	(1,1)	(8,1)

**Table 5:** *α* ratio for cell-type trees on H3K4me3 (replicate 2) data.

	Epithelial (9)	Fibroblast (12)
ML tree	$\frac{6}{6}$, $\frac{7}{16}$, $\frac{8}{22}$, $\frac{9}{26}$	$\frac{8}{8}$, $\frac{10}{12}$, $\frac{11}{15}$, $\frac{12}{17}$
MLQA tree	$\frac{7}{7}$, $\frac{8}{14}$, $\frac{9}{28}$	$\frac{3}{3}$, $\frac{6}{7}$, $\frac{11}{13}$, $\frac{12}{17}$
IQA tree	$\frac{4}{4}$, $\frac{8}{17}$, $\frac{9}{28}$	$\frac{6}{6}$, $\frac{7}{8}$, $\frac{11}{13}$, $\frac{12}{17}$

In terms of grouping the 9 Epithelial cell-types, MLQA is better than IQA and ML since it placed 7 of them in a single cluster. Likewise, for grouping 8 Fibroblast cell-types, MLQA took substantially less number of cell-types compared to ML (14 for MLQA and 22 for ML tree). However, when we considered all the 9 Epithelial cell-types, ML and IQA achieved better *α* values than MLQA.

### H3K27me3 dataset

Histone H3 lysine 27 trimethylation (H3K27me3) is an important epigenetic mark which is associated with the downregulation of genes [40]. Thus, it acts in opposition to H3K4me3 which is associated with gene activation [41]. Replicate 1 from this dataset has 20 cell-types which contains both the normal and cancerous cell-types and replicate 1 and 2 together has 37 cell-types.

#### Replicate 1

**Fig 7:**
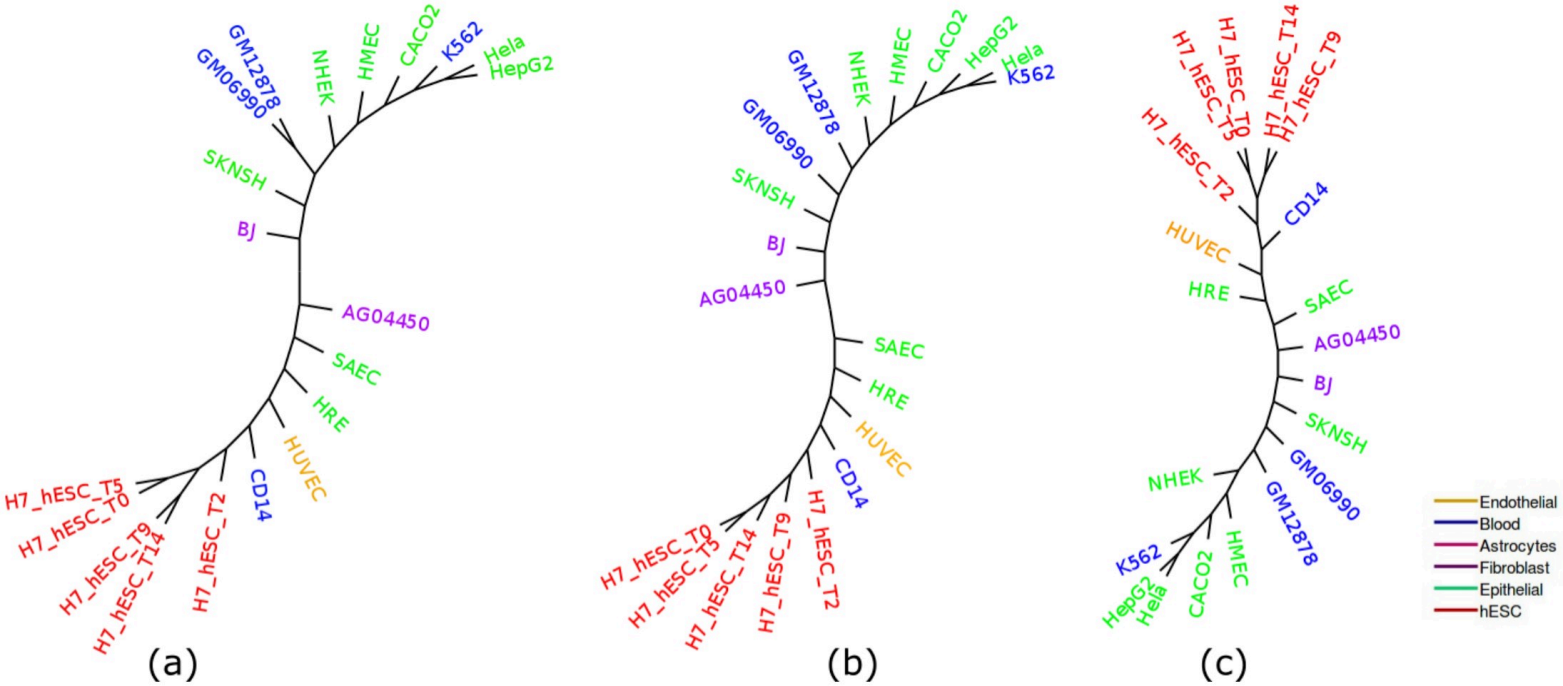
Cell-type trees on H3K27me3 (replicate 1). (a) IQA approach, (b) MLQA approach, and (c) ML approach.

Fig 7 shows the ML, IQA and MLQA estimated trees on the 5 cell-type groups from replicate 1. All these methods are comparable in terms of grouping various cell-types. IQA tree is better than ML and MLQA since it did a better job in grouping the Blood and Epithelial cell-types (see Table 6). All these methods performed poorly on the Epithelial cell-types as the size of the largest cluster with only the Epithelial cell-types is 2. Table 7 shows the similarity among these three approaches in terms of the *α* ratio for Epithelial group. Note that all these methods take a subtree with 9 cell-types to group 6 (out of 8) Epithelial cell-types. However, to accommodate all the 8 cell-types, the size of the subtree was increased to 13.

**Table 6:** Groupings for cell-type trees on H3K27me3 (replicate 1) data.

	hESC (5)	Epithelial (8)	Fibroblast (2)	Blood (4)	Endothelial (1)
ML tree	(5,0)	(2,1)	(1,1)	(1,1)	(1,0)
MLQA tree	(5,0)	(1,1)	(1,1)	(1,1)	(1,0)
IQA tree	(5,0)	(2,1)	(1,1)	(2,1)	(1,0)

**Table 7:** *α* ratio for cell-type trees on H3K27me3 (replicate 1) data.

	Epithelial (8)
ML tree	$\frac{2}{2}$, $\frac{5}{6}$, $\frac{6}{9}$, $\frac{8}{13}$
MLQA tree	$\frac{1}{1}$, $\frac{5}{6}$, $\frac{6}{9}$, $\frac{8}{13}$
IQA tree	$\frac{2}{2}$, $\frac{5}{6}$, $\frac{6}{9}$, $\frac{8}{13}$

**Fig 8:**
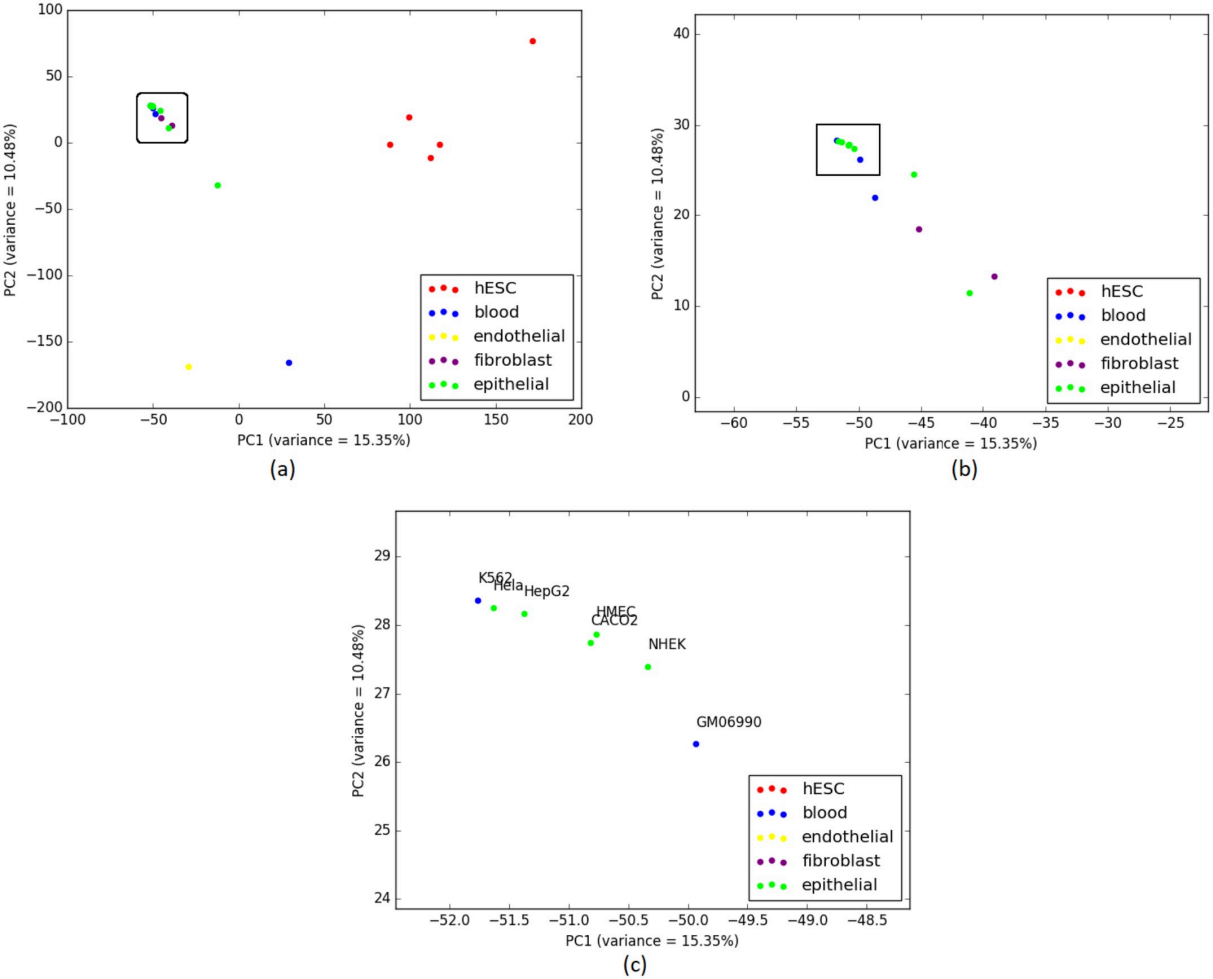
PCA on H3K27me3 (replicate 1) and corresponding scores are plotted on PC1 and PC2. (a) 12 closely clustered cell-types are selected from the 20 cell-types in a rectangular box. (b) 12 cell-types from (a) are zoomed in for a comprehensible view which helps us note that these 12 includes 2 Fibroblast cell-types just like the cell-type trees from all three approaches in Fig 7. Here 2 Fibroblast cell-types (BJ and AG04450) are located close to the cluster of 3 Blood cell-types and 8 Epithelial cell-types. 7 cell-types from these 12 are similarly enclosed in a rectangle for further investigation. (c) 7 cell-types from (b) are zoomed in and annotated where we can see 2 Blood cell-types (K562 and GM06990) are closely related to 5 Epithelial cell-types which is a nearly similar scenario for the corresponding trees as well.

We can see from Table 6 that IQA approach outperforms the other two on the cell-types from Blood (the largest cluster in IQA tree contains 2 cell-types whereas the other two trees contain only 1 cell-type). Likewise, for Epithelial group, IQA and ML based approaches perform slightly better than the MLQA approach. For the remaining groups, these three approaches show identical clustering. PCA analysis on this data is shown in Fig 8 which strongly support our findings from the cell-type trees estimated by various approaches.

#### Replicate 1 and 2

**Fig 9:**
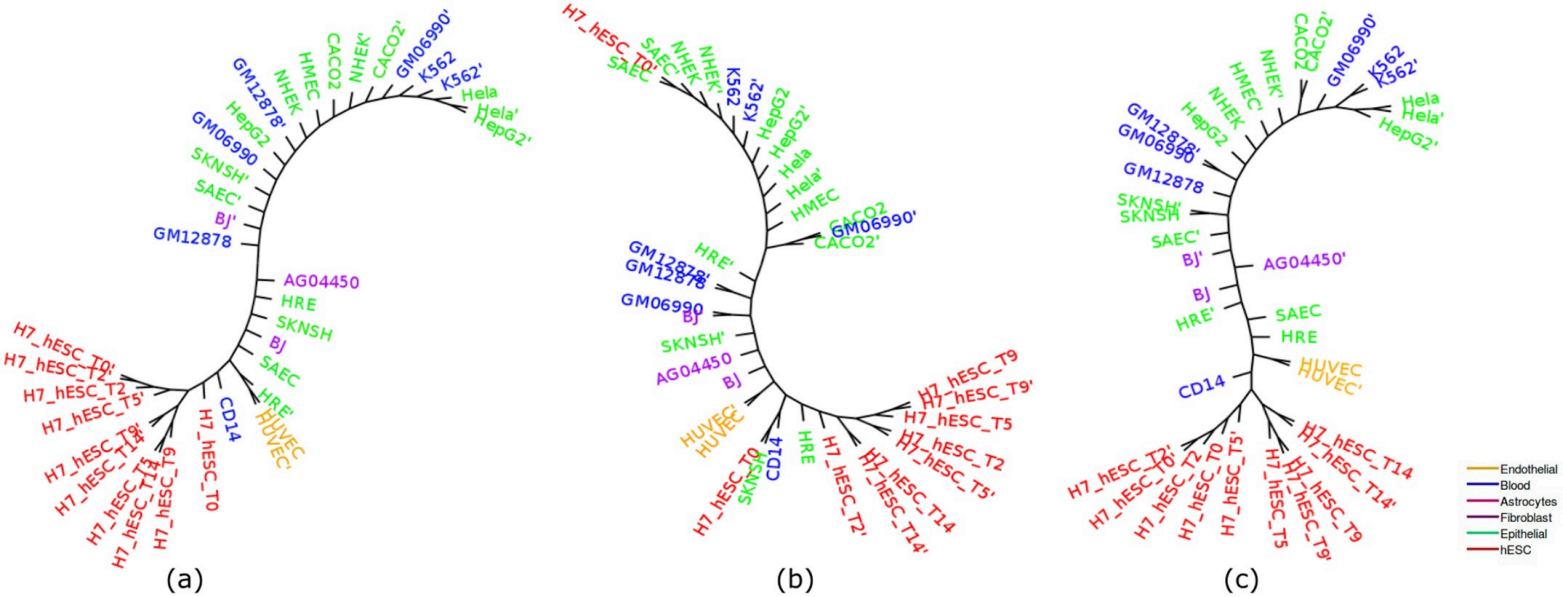
Cell-type trees on H3K27me3 (replicate 1 and 2). (a) IQA approach, (b) MLQA approach and (c) ML approach.

IQA and ML trees are very similar except that ML tree is slightly better on the Blood cell-types. The MLQA approach reconstructs a slightly worse tree in terms of grouping the Epithelial cell-types (see Fig 9 and Table 8). The other two methods (IQA and ML) also performed poorly on Epithelial cell-types as they were able to cluster only 3 cell-types in the largest cluster.

**Table 8:** Groupings for cell-type trees on H3K27me3 (replicate 1 and 2) data.

	hESC (10)	Epithelial (15)	Fibroblast (3)	Blood (7)	Endothelial (2)
ML tree	(10,0)	(3,2)	(1,1)	(2,2)	(2,0)
MLQA tree	(8,1)	(1,1)	(1,1)	(2,1)	(2,0)
IQA tree	(10,0)	(3,1)	(1,1)	(1,1)	(2,0)

Fig 9 shows that while IQA approach succeeds in clustering hESC and Endothelial cell-types, ML based approach showed slightly better performance for Epithelial and Blood cell-types. MLQA estimated tree is comparatively worse than the other two trees. All the approaches exhibited poor performance on the Epithelial group. There are 15 cell-types in this group but all the approaches were able to cluster only 3 cell-types in the largest subtree.

**Fig 10:**
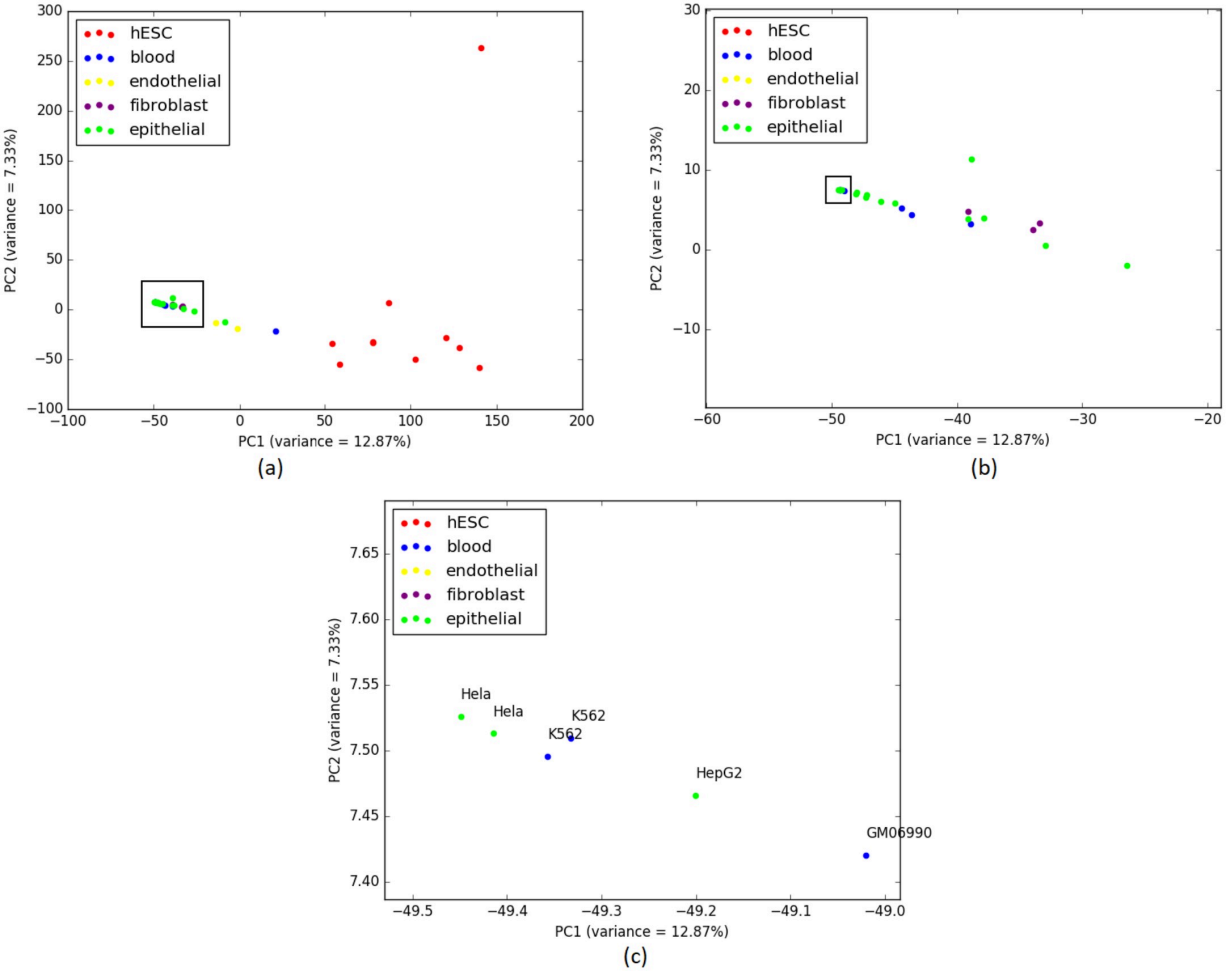
PCA on H3K27me3 (replicate 1 and 2) and corresponding scores are plotted on PC1 and PC2. (a) 23 closely clustered cell-types including Blood, Epithelial and Fibroblast cell-types are selected from the 37 cell-types in a rectangular box. (b) Upon zooming in the 23 cell-types from (a), we can see that 3 Fibroblast cell-types (BJ(1), BJ(2) and AG04450) have entered into the cluster comprising Epithelial and Blood cell-types which is an identical situation to the clustering tendencies exhibited in the cell-type trees in Fig 9. 6 cell-types from these 23 are similarly enclosed in a rectangle for further investigation. (c) 6 cell-types from (b) are zoomed in and annotated. Close relationships among the the cancerous cell-types from Epithelial group (Hela(1), Hela(2) and HepG2) and the cancerous cell-types from Blood group (K562(1), K562(2) and GM06990) are suggested by both the PCA plot and the cell-type trees.

Table 9 shows *α* ratio for Epithelial group and it is noteworthy that a similar performance degradation (as we observed on Replicate 1) was observed when we considered all the cell-types. For 14 cell-types of this group, IQA approach provides the smallest *α* value. Yet, while adding the 15^th^, ML based approach achieved a smaller value of *α* than IQA and MLQA. PCA analyses are demonstrated in Fig 10 which support the findings from the cell-type trees. We note that the cancerous cell-types from Epithelial group (Hela(1), Hela(2) and HepG2) and Blood group (K562(1), K562(2) and GM06990) are closely related both in the cell-type trees and in the PCA plot.

**Table 9:** *α* ratio for cell-type trees on H3K27me3 (replicate 1 and 2) data.

	Epithelial (15)
ML tree	$\frac{3}{3}$, $\frac{9}{12}$, $\frac{12}{18}$, $\frac{15}{24}$
MLQA tree	$\frac{1}{1}$, $\frac{4}{5}$, $\frac{9}{12}$, $\frac{12}{16}$, $\frac{13}{21}$, $\frac{15}{29}$
IQA tree	$\frac{3}{3}$, $\frac{8}{11}$, $\frac{9}{13}$, $\frac{11}{16}$, $\frac{13}{21}$, $\frac{14}{23}$, $\frac{15}{26}$

### Comparative analyses on H3k4me3 and H3K27me3

We compared the trees estimated on H3K4me3 with the trees estimated on H3K27me3. We observed that the relationships between various cell-types are consistent between these two dataset. Similar cell-types tend to group together with a few exceptions (as described in previous sections). Moreover, cell-types from the same tissue type tend to form clusters on both H3k4me3 and H3K27me3. The similarity of results between the two dataset reinforces our opinion and the results from previous studies [1, 6, 13] that phylogenetic analyses yield biologically meaningful results on such data.

### H3K36me3 dataset

**Fig 11:**
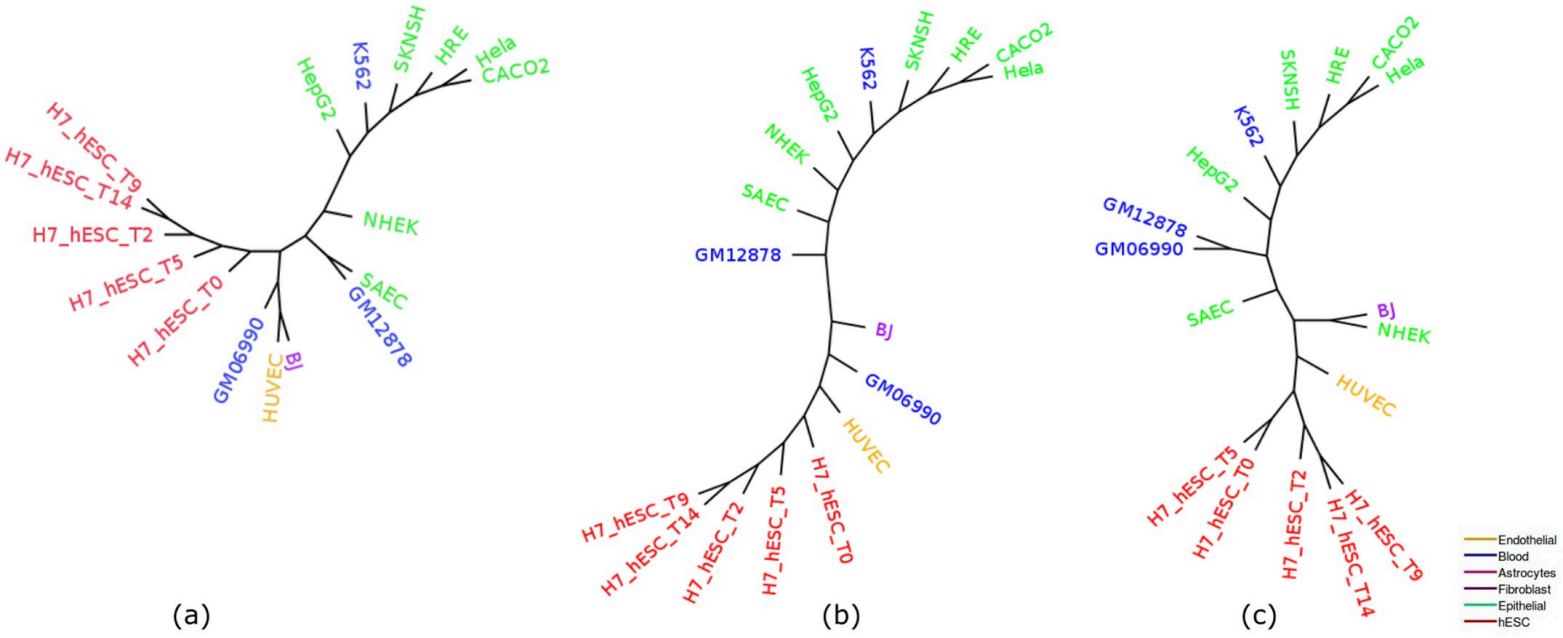
Cell-type trees on H3K36me3 (replicate 1). (a) IQA approach, (b) MLQA approach, and (c) ML based approach.

**Fig 12:**
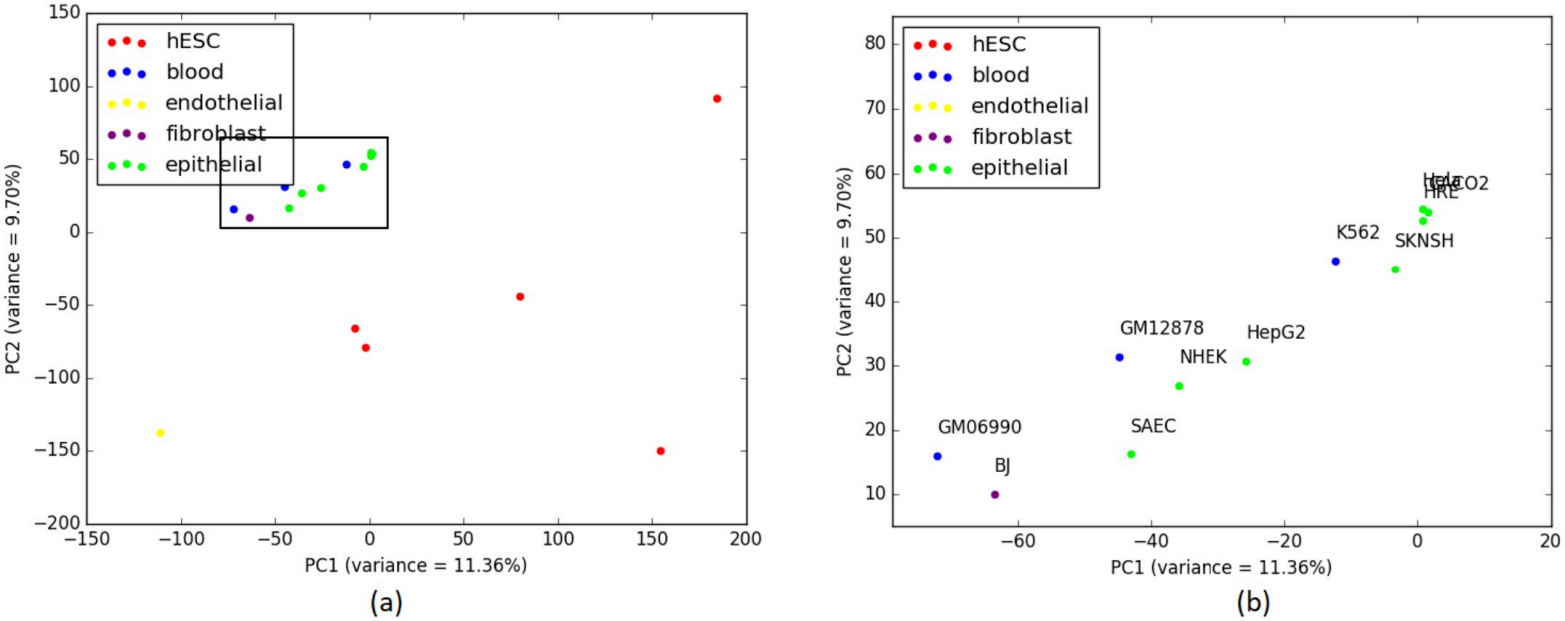
PCA on H3K36me3 (replicate 1) and corresponding scores are plotted on PC1 and PC2. (a) 11 closely clustered cell-types that include Blood, Epithelial and Fibroblast are selected from the 17 cell-types in a rectangular box. (b) When these 11 cell-types from (a) are zoomed in, it becomes evident that BJ from Fibroblast and GM06990, GM12878 and K562 from Blood are intruders in the Epithelial cluster which is a comparable scenario with the Epithelial clusters from the cell-type trees in Fig 11.

H3K36me3 (Histone H3 lysine 36 methylation) is a histone modification involved in epigenetic regulation and is a common epigenetic mark [42]. The modifications of H3K36 are very diverse and play roles in many important biological processes such as DNA replication, transcription, recombination and repair of DNA damage [43]. This dataset (replicate 1) includes 17 cell-types. Fig 11 shows the trees estimated by ML, IQA and MLQA. The performance metrics are shown in Tables 10 and 11. PCA plot is shown in Fig 12. In general, the relative performance of these three methods are similar to what we observed on the previous two datasets (H3K4me3 and H3K27me3).

**Table 10:** Groupings for cell-type trees on H3K36me3 (replicate 1) data.

	hESC (5)	Epithelial (7)	Fibroblast (1)	Blood (3)	Endothelial (1)
ML tree	(5,0)	(4,1)	(1,0)	(2,1)	(1,0)
MLQA tree	(5,0)	(4,1)	(1,0)	(1,1)	(1,0)
IQA tree	(5,0)	(4,1)	(1,0)	(1,1)	(1,0)

**Table 11:** *α* ratio for cell-type trees on H3K36me3 (replicate 1) data.

	Epithelial (7)
IQA tree	$\frac{4}{4}$, $\frac{6}{7}$, $\frac{7}{9}$
MLQA tree	$\frac{4}{4}$, $\frac{7}{8}$
ML tree	$\frac{4}{4}$, $\frac{5}{6}$, $\frac{6}{9}$, $\frac{7}{11}$

### H3K27ac dataset

**Fig 13:**
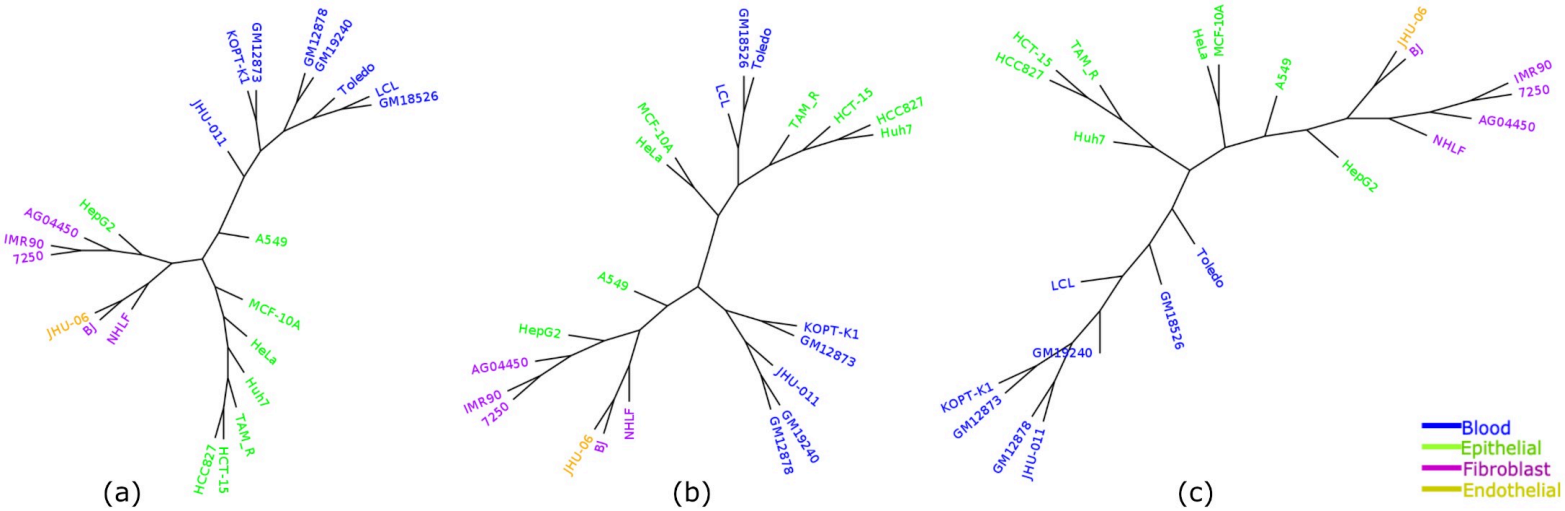
Cell-type trees for H3K27ac. (a) IQA approach (b) MLQA approach and (c) ML approach.

Enhancer is an active regulatory element in genome which can affect gene transcription [44]. We analyzed H3K27ac as it is an important enhancer mark and computed cell-type trees using IQA, MLQA and ML based approaches (Fig 13). This dataset contains 22 cells of 4 different types (Epithelial, Fibroblast, Blood and Endothelial). The performance of various methods on H3K27ac dataset is demonstrated in Tables 12 and 13 and Fig 13. The experimental results show that, similar to our observation on other dataset, the proposed phylogenetic approaches can construct meaningful trees on histone acetylation data since they tend to cluster the similar cell-types together. Table 12 reflects the fact that IQA tree clusters Epithelial cell-lines better than the other two approaches. MLQA tree fails to cluster all Blood cell-lines in one sub-tree devoid of any alien cell-lines. ML based approach performed slightly better than IQA and MLQA on Fibroblas cell-types as the largest Fibroblast clade in ML tree contains 4 cell-types, whereas for IQA and MLQA approaches, this value is 3. Table 13 shows the alpha ratio for Epithelial and Fibroblast groups. We have not included Blood in this table since both IQA and ML approaches were able to cluster this group in an ideal manner.

**Table 12:** Groupings for cell-type trees on H3K27ac data.

	Epithelial (8)	Fibroblast (5)	Blood (8)	Endothelial (1)
ML tree	(4,2)	(4,1)	(8,0)	(1,0)
MLQA tree	(4,2)	(3,1)	(5,3)	(1,0)
IQA tree	(6,1)	(3,1)	(8,0)	(1,0)

**Table 13:** *α* ratio for cell-type trees on H3K27ac data.

	Epithelial (8)	Fibroblast (5)
IQA tree	$\frac{6}{6}$, $\frac{8}{14}$	$\frac{3}{3}$, $\frac{5}{7}$
MLQA tree	$\frac{4}{4}$, $\frac{6}{9}$, $\frac{8}{17}$	$\frac{3}{3}$, $\frac{5}{7}$
ML tree	$\frac{4}{4}$, $\frac{8}{14}$	$\frac{4}{4}$, $\frac{5}{6}$

### Combined analyses with H3k4me3 and H3K27me3

Combined analyses (also known as concatenation) is a traditional approach to species tree (a phylogenetic tree showing the evolutionary history of a group of species) estimation from multi-locus data. Combined analyses concatenates gene sequence alignments into a supergene matrix, and then estimates the species tree using a sequence based tree estimation technique (e.g., maximum parsimony, maximum likelihood, Bayesian analysis, etc.). Although combined analyses is not statistically consistent [45] and can return inaccurate trees with high confidence [46–49], it has been used in many biological studies since it can construct highly accurate species trees by leveraging the high amount of phylogenetic signal from the combined supermatrix, especially when the degree of gene tree discordance is low [50, 51]. To demonstrate the applicability of combined analyses and thereby showing the feasibility of analyzing multiple egigenetic marks at the same time, we performed combined analyses on H3k4me3 and H3K27me3 data.

We selected these two epigenetic marks since they have the highest number of common cell-lines among the datasets that we have analysed in this study. There are 13 cells common to both these dataset. We combined the alignments containing 13 cell-lines, resulting from the overlapping representations of H3k4me3 and H3K27me3, into a supermatrix and analyzed the data using maximum likelihood and quartet based techniques.

**Fig 14:**
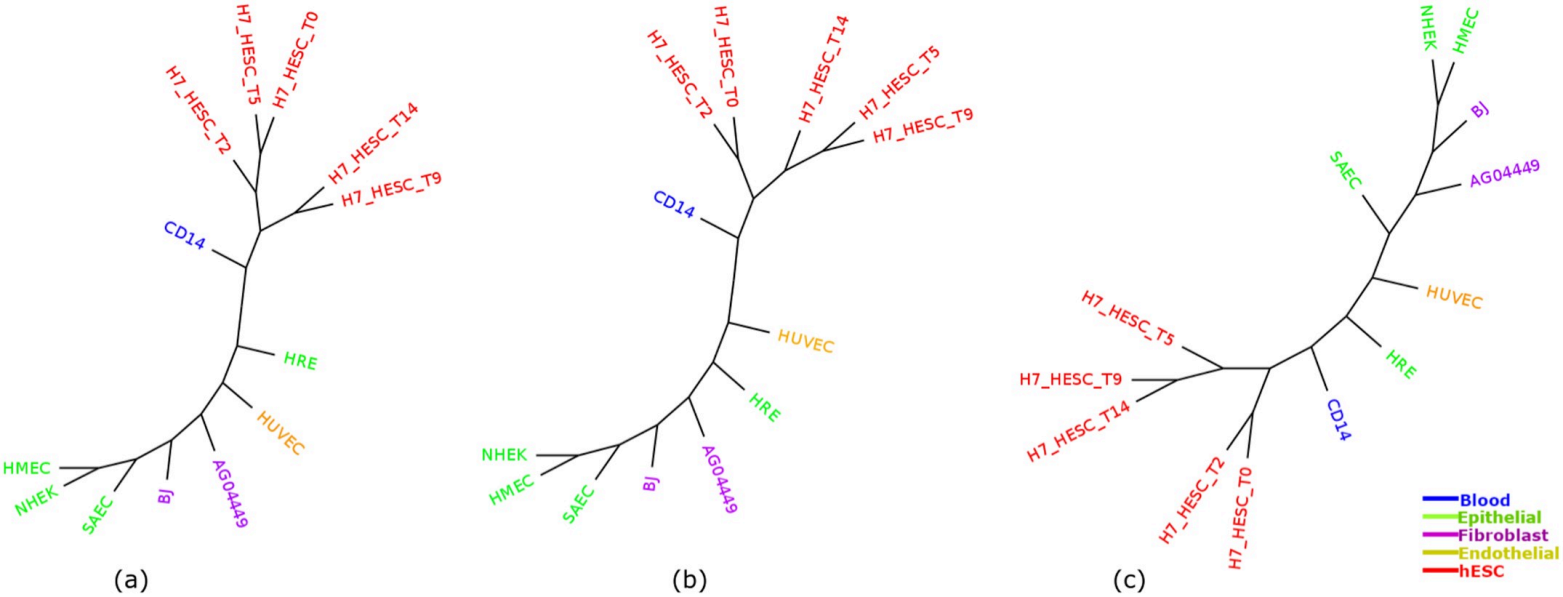
Cell-type trees estimated using combined analyses on H3K4me3 and H3K27me3. (a) IQA approach, (b) MLQA approach, and (c) ML based approach.

We selected 13 common cell-types from these two marks and computed cell-type tree based on three approaches (IQA, MLQA and ML). The results from combined analyses show that the cell-type trees constructed from merged peak data of the same cell-lines from these two epigenetic marks appear to carry a meaningful clustering pattern. We can see from Fig 14 that, 5 cell-lines from hESC are always clustered together which is similar to the behavior of hESC clusters from the previous cell-type trees computed from individual epigenetic mark. Also, Tables 14 and 15 reflect the fact that, IQA and MLQA approaches outperforms ML based approach while clustering Epithelial cell-lines. For other cell-types, performance of all these methods are similar.

**Table 14:** Groupings for cell-type trees estimated by combined analyses on H3K4me3 and H3K27me3.

	hESC (5)	Epithelial (4)	Fibroblast (2)	Blood (1)	Endothelial (1)
ML tree	(5,0)	(2,1)	(1,1)	(1,0)	(1,0)
MLQA tree	(5,0)	(3,1)	(1,1)	(1,0)	(1,0)
IQA tree	(5,0)	(3,1)	(1,1)	(1,0)	(1,0)

**Table 15:** *α* ratio for cell-type trees estimated by combined analyses on H3K4me3 and H3K27me3.

	Epithelial (4)	Fibroblast (2)
IQA tree	$\frac{3}{3}$, $\frac{4}{7}$	$\frac{1}{1}$, $\frac{2}{5}$
MLQA tree	$\frac{3}{3}$, $\frac{4}{6}$	$\frac{1}{1}$, $\frac{2}{5}$
ML tree	$\frac{2}{2}$, $\frac{3}{5}$, $\frac{4}{7}$	$\frac{1}{1}$, $\frac{2}{4}$

These results demonstrate the feasibility of applying combined anslysis on multiple epigenetic marks. Although epigenetic marks can be modified relatively independently from each other which is similar to the independent evolution of multiple markers (genes) within a group of species [52], combined analyses on multiple epigenetic marks can be useful to elucidate the relationships among various cell-types.

## Conclusions

We proposed two quartet-based phylogenetic tree construction methods to infer cell differentiation trees. Due to the growing awareness that phylogenetic tree estimation methods are useful in inferring processes of cell differentiation, various standard phylogenetic methods have been applied on various epigenetic information. The results of this study supported the validity of quartet-based approach (which is being widely used in constructing species trees from multi-locus data) for inferring reliable cell-type trees using ChIP-Seq histone modification data. We analyzed a collection of real biological data, containing both normal and cancerous cell-types with multiple replicates, to assess the performance of our proposed methods. Experimental results suggest that our methods can reconstruct meaningful cell-type trees. In this study, we also proposed a new metric to evaluate the reliability of cell-type trees.

Phylogenetic methods, in most of the cases, were successfully able to place similar cell-types together within a clade, but we observed a few cases (especially for Epithelial cell-types) where different cell-types from different groups were clustered together. PCA analyses of the overlap representation of the ChIP-Seq histone modification data suggest that this is possibly not due to any shortcomings of the phylogenetic tree estimation methods, rather the underlying data may not have sufficient information to clearly distinguish these cell-types. It could also be due to the close interaction and transition between cell-types [53–55]. For example, Epithelial cells can give rise to Fibroblasts under certain conditions, which is known as epithelial-mesenchymal transition (EMT) [56, 57]. The reverse phenomenon, where Fibroblasts may give rise to Epithelial, a process called mesenchymal–epithelial transition (MET), is also possible [58, 59]. However, more rigorous experiments are required to further validate these hypotheses regarding the presence of different cell-types within the subtree of a particular group.

This study shows the strength and applicability of standard phylogenetic tree estimation techniques in supplementing the traditional laborious *in vitro* experiments for elucidating the relationships among various cell-types. Thus, we believe that the approaches presented in this study will help biologists and systematists to address various fundamental questions in cell development and differentiation. However, this study can be extended in several directions. Our proposed methods are applicable to other epigenetic marks (e.g., RNA-seq) as well given that we have an appropriate data representation (e.g., window and overlap representation) technique so we can apply various phylogenetic methods. Kin *et al*. [1] converted the expression data into qualitative data (expressed/non-expressed) and applied maximum parsimony based phylogenetic tree estimation method to construct cell-type trees from RNA-Seq data. Similarly, our techniques can be applied to the RNA-Seq data to infer cell differentiation trees. It would be interesting to analyze how various phylogenetic approaches perform on RNA-Seq data and to investigate what types of data representations are appropriate for various epigenetic marks. We leave this as a future work. Another important avenue is to investigate how to remove “batch effects”—the systematic error introduced when samples are processed in multiple batches. The batch effect may mislead the phylogenetic analysis, because it can inflate the correlations within the same batch [60]. However, it remains unclear to what extent batch effect may influence the phylogenetic analyses of the Chip-Seq data, and appropriate extensive simulation studies need to be designed and performed to better understand the impact of batch effects. One approach for eliminating the risk of batch effects is to perform the whole study in a single batch [60]. More sophisticated methods (for an example, principal variation component analysis (PVCA) [61, 62]) can be used to measure how much variation in the data is due to batch effects. Finally, proposing theoretical framework and mathematical basis for comparing various phylogenetic approaches for estimating cell-differentiation trees using epigenetic data would be important to understand the relative performance of various techniques under different realistic model conditions.
